# Data on unsafe riding behaviors among 1960 shared bicycle riders in urban China

**DOI:** 10.1016/j.dib.2019.104329

**Published:** 2019-07-27

**Authors:** Xiaolin Wu, Wangxin Xiao, Conghui Deng, David C. Schwebel, Guoqing Hu

**Affiliations:** a Zhou Enlai School of Government, Nankai University, Tianjin, 300071, China; b Department of Epidemiology and Health Statistics, Xiangya School of Public Health, Central South University, Changsha, 410078, Hunan, China; c Department of Administration Management, School of Public Administration, Central South University, Changsha, 410083, Hunan, China; d Department of Psychology, University of Alabama at Birmingham, Birmingham, AL, 35294, USA

**Keywords:** Shared bicycle, Riding behaviors, China, Urban area

## Abstract

This data article quantifies the extent of shared bicycle riding risks for shared-bicycle riders in urban China. The data were collected through a WeChat-based online survey, with a valid sample of 1960 respondents. It reports the basic descriptive statistics through eight tables concerning various unsafe shared bicycle riding behaviors, and complete frequency data from riders concerning eight unsafe riding behaviors. The data can be used for comparisons with other studies using the same outcome measures, which are valuable to generate specialized and targeted solutions to reduce unsafe riding behaviors. For further information, please refer to the full article entitled “Unsafe riding behaviors of shared-bicycle riders in urban China: A retrospective survey”.(Wu et al., 2019).

**Table:** Specifications Table

Subject	*Public health and health policy*
Specific subject area	*Road traffic injury (RTI)*
Type of data	*Tables*
How data were acquired	*Self-report survey from researcher-designed electronic questionnaire*
Data format	*Raw, filtered, analyzed, descriptive statistical data*
Parameters for data collection	*Sample consisted of shared-bicycle users in urban China. The researchers used an electronic questionnaire to investigate whether participants self-report engaging in eight unsafe shared-bicycle riding behaviors in the past month.*
Description of data collection	*We estimated a priori experimental needs for a one-month survey period and used an iterative sampling through a “snowball technique” recruitment strategy through a WeChat-based online survey for a month, from September 7, 2017 to October 6, 2017.*
Data source location	*Urban China*
Data accessibility	*Data are accessible with the article*
Related research article	*The associated research article to this data set is*[1]*.*

**Table:** 

**Value of the data** • The data provide the first published epidemiological report about eight unsafe bicycle riding behaviors and basic characteristics from a sample of 1960 shared bicycle riders in urban China. • The data allow anyone to duplicate the results of comparisons for each behavior across sex, age, education, city of shared bicycle use and shared bicycle travel-related information. • The data could be used for comparisons with other studies using the same or similar outcome measures.

## 1 Data

Table 1, Table 2, Table 3, Table 4, Table 5, Table 6, Table 7, Table 8 show the complete frequencies of eight unsafe riding behaviors: not wearing helmets, running red lights, cycling against the traffic flow, riding in a motor vehicle lane, riding in a pedestrian lane, carrying passengers, using a cell phone while riding and eating while riding, among 1960 surveyed shared-bicycle riders in the past month in urban China. The data allow researchers to conduct further analyses for specific research purposes. The sample had a mean age of 27.63 years (standard deviation: 9.50 years).

**Table 1:** Proportion of riders not wearing helmets.

Variable	Number (%)	Frequency of behavior (%)			
		Always	Often	Sometimes	Never
Total	1960 (100)	95.4	2.2	0.9	1.5
Sex					
Male	833 (43)	93.2	3.1	1.3	2.4
Female	1127 (58)	97.0	1.6	0.5	0.9
Age group					
≤25 years	1056 (54)	97.3	1.8	0.5	0.5
26∼35 years	623 (32)	94.9	2.4	1.0	1.8
≥36 years	281 (14)	89.3	3.6	2.1	5.0
Level of education					
Postgraduate or higher	769 (39)	97.4	1.6	0.4	0.7
Undergraduate	970 (50)	96.5	2.0	0.7	0.8
All others	221 (11)	83.3	5.9	3.2	7.7
Type of urban area of bicycle use					
Central municipality	450 (23)	97.8	0.7	0.4	1.1
Provincial capital	1092 (56)	96.9	1.7	0.6	0.7
Deputy provincial city	90 (5)	93.3	3.3	1.1	2.2
All others	328 (17)	87.5	5.8	2.1	4.6
Province/City of bicycle use					
Hunan	579 (30)	95.2	2.2	1.0	1.6
Guangdong	230 (12)	96.1	1.7	0.9	1.3
Beijing	164 (8)	98.8	0.0	0.6	0.6
Tianjin	144 (7)	98.6	1.4	0.0	0.0
All others	843 (43)	94.1	3.0	0.9	2.0
Reason for travel					
Commuting to work/school	1070 (55)	97.6	1.1	0.5	0.8
Entertainment	544 (28)	94.9	3.3	0.7	1.1
Physical exercise	180 (9)	82.8	7.2	3.3	6.7
Others	166 (9)	96.4	0.6	1.2	1.8
Riding hours per week					
<1 hour	240 (12)	98.8	0.4	0.0	0.8
1–2 hours	732 (37)	97.5	1.4	1.0	0.1
3–5 hours	877 (45)	93.5	3.0	0.8	2.7
>5 hours	111 (6)	88.3	6.3	2.7	2.7
Type of typical riding days					
Weekday	1105 (56)	97.2	1.4	0.6	0.7
Weekend or holiday	855 (44)	93.0	3.3	1.2	2.6
Typical riding time					
Morning rush hours	399 (20)	93.7	2.5	1.3	2.5
Evening rush hours	698 (36)	96.3	1.9	0.7	1.1
Other times	863 (44)	95.4	2.4	0.8	1.4

**Table 2:** Proportion of riders running red lights.

Variable	Number (%)	Frequency of behavior (%)			
		Always	Often	Sometimes	Never
Total	1960 (100)	0.5	1.4	18.3	79.8
Sex					
Male	833 (43)	0.5	1.9	22.3	75.3
Female	1127 (58)	0.5	1.1	15.3	83.1
Age group					
≤25 years	1056 (54)	0.3	1.4	18.1	80.2
26∼35 years	623 (32)	0.5	1.3	19.4	78.8
≥36 years	281 (14)	1.4	1.8	16.4	80.4
Level of education					
Postgraduate or higher	769 (39)	0.7	1.6	20.5	77.2
Undergraduate	970 (50)	0.2	1.4	16.7	81.6
All others	221 (11)	1.4	0.9	17.2	80.5
Type of urban area of bicycle use					
Central municipality	450 (23)	0.7	0.9	24.0	74.4
Provincial capital	1092 (56)	0.2	1.5	17.1	81.2
Deputy provincial city	90 (5)	1.1	1.1	14.4	83.3
All others	328 (17)	1.2	2.1	15.2	81.4
Province/City of bicycle use					
Hunan	579 (30)	0.0	1.7	15.7	82.6
Guangdong	230 (12)	0.4	0.4	20.9	78.3
Beijing	164 (8)	1.2	1.2	31.7	65.9
Tianjin	144 (7)	0.0	0.7	15.3	84.0
All others	843 (43)	0.8	1.7	17.2	80.3
Reason for travel					
Commuting to work/school	1070 (55)	0.4	1.7	22.1	75.8
Entertainment	544 (28)	0.4	1.5	11.9	86.2
Physical exercise	180 (9)	1.7	1.1	16.1	81.1
Others	166 (9)	0.6	0.0	16.3	83.1
Riding hours per week					
<1 hour	240 (12)	0.0	0.8	14.2	85.0
1–2 hours	732 (37)	0.4	1.5	17.9	80.2
3–5 hours	877 (45)	0.2	1.6	19.3	78.9
>5 hours	111 (6)	4.5	0.9	21.6	73.0
Type of typical riding days					
Weekday	1105 (56)	0.5	1.6	21.6	76.3
Weekend or holiday	855 (44)	0.6	1.2	13.9	84.3
Typical riding time					
Morning rush hours	399 (20)	0.3	1.8	23.8	74.2
Evening rush hours	698 (36)	0.7	1.9	16.5	80.9
Other times	863 (44)	0.5	0.9	17.1	81.5

**Table 3:** Proportion of riders cycling against the traffic flow.

Variable	Number (%)	Frequency of behavior (%)			
		Always	Often	Sometimes	Never
Total	1960 (100)	0.8	2.6	42.0	54.5
Sex					
Male	833 (43)	1.2	3.6	45.6	49.6
Female	1127 (58)	0.5	1.9	39.4	58.2
Age group					
≤25 years	1056 (54)	0.5	2.6	40.5	56.4
26∼35 years	623 (32)	0.8	2.9	45.9	50.4
≥36 years	281 (14)	2.1	2.1	39.1	56.6
Level of education					
Postgraduate or higher	769 (39)	0.5	2.9	45.0	51.6
Undergraduate	970 (50)	0.7	2.5	41.9	54.9
All others	221 (11)	2.3	2.3	32.6	62.9
Type of urban area of bicycle use					
Central municipality	450 (23)	0.9	2.9	51.3	44.9
Provincial capital	1092 (56)	0.5	2.3	41.8	55.4
Deputy provincial city	90 (5)	0.0	4.4	33.3	62.2
All others	328 (17)	1.8	2.7	32.6	62.8
Province/City of bicycle use					
Hunan	579 (30)	0.3	2.2	40.9	56.5
Guangdong	230 (12)	0.9	3.0	41.3	54.8
Beijing	164 (8)	1.2	4.9	64.6	29.3
Tianjin	144 (7)	0.7	1.4	35.4	62.5
All others	843 (43)	1.1	2.5	39.7	56.7
Reason for travel					
Commuting to work/school	1070 (55)	0.8	3.4	46.3	49.5
Entertainment	544 (28)	0.7	1.5	35.7	62.1
Physical exercise	180 (9)	1.7	2.2	33.3	62.8
Others	166 (9)	0.0	1.8	45.2	53.0
Riding hours per week					
<1 hour	240 (12)	0.4	2.5	42.1	55.0
1–2 hours	732 (37)	0.7	3.0	43.3	53.0
3–5 hours	877 (45)	0.6	2.1	41.5	55.9
>5 hours	111 (6)	4.5	4.5	37.8	53.2
Type of typical riding days					
Weekday	1105 (56)	0.7	3.3	45.7	50.2
Weekend or holiday	855 (44)	0.9	1.6	37.3	60.1
Typical riding time					
Morning rush hours	399 (20)	0.5	5.5	45.1	48.9
Evening rush hours	698 (36)	1.1	1.6	41.7	55.6
Other times	863 (44)	0.7	2.1	40.9	56.3

**Table 4:** Proportion of riders riding in a motor vehicle lane.

Variable	Number (%)	Frequency of behavior (%)			
		Always	Often	Sometimes	Never
Total	1960 (100)	1.7	4.4	42.0	51.9
Sex					
Male	833 (43)	2.0	5.2	46.2	46.6
Female	1127 (58)	1.4	3.8	39.0	55.8
Age group					
≤25 years	1056 (54)	1.7	5.2	43.7	49.4
26∼35 years	623 (32)	1.3	3.9	42.5	52.3
≥36 years	281 (14)	2.5	2.5	34.9	60.1
Level of education					
Postgraduate or higher	769 (39)	1.4	3.8	43.8	51.0
Undergraduate	970 (50)	1.5	4.8	43.3	50.3
All others	221 (11)	3.2	4.5	30.3	62.0
Type of urban area of bicycle use					
Central municipality	450 (23)	2.0	2.9	39.1	56.0
Provincial capital	1092 (56)	1.5	4.7	44.5	49.4
Deputy provincial city	90 (5)	1.1	5.6	52.2	41.1
All others	328 (17)	2.1	5.2	35.1	57.6
Province/City of bicycle use					
Hunan	579 (30)	1.9	6.6	47.2	44.4
Guangdong	230 (12)	0.9	6.1	50.0	43.0
Beijing	164 (8)	1.2	2.4	49.4	47.0
Tianjin	144 (7)	2.8	3.5	30.6	63.2
All others	843 (43)	1.7	3.0	36.9	58.5
Reason for travel					
Commuting to work/school	1070 (55)	1.7	5.0	44.6	48.8
Entertainment	544 (28)	1.3	3.5	40.8	54.4
Physical exercise	180 (9)	3.9	2.8	30.0	63.3
Others	166 (9)	0.6	5.4	42.8	51.2
Riding hours per week					
<1 hour	240 (12)	1.2	3.8	43.3	51.7
1–2 hours	732 (37)	1.2	4.4	43.3	51.1
3–5 hours	877 (45)	1.6	4.4	40.7	53.2
>5 hours	111 (6)	6.3	5.4	41.4	46.8
Type of typical riding days					
Weekday	1105 (56)	2.0	5.0	43.4	49.6
Weekend or holiday	855 (44)	1.3	3.6	40.2	54.9
Typical riding time					
Morning rush hours	399 (20)	2.3	4.5	43.1	50.1
Evening rush hours	698 (36)	1.6	4.3	42.4	51.7
Other times	863 (44)	1.5	4.4	41.3	52.8

**Table 5:** Proportion of riders riding in a pedestrian lane.

Variable	Number (%)	Frequency of behavior (%)			
		Always	Often	Sometimes	Never
Total	1960 (100)	7.9	17.1	52.1	23.0
Sex					
Male	833 (43)	7.1	19.6	53.4	19.9
Female	1127 (58)	8.4	15.3	51.1	25.2
Age group					
≤25 years	1056 (54)	7.4	17.1	51.9	23.6
26∼35 years	623 (32)	6.3	18.3	53.1	22.3
≥36 years	281 (14)	13.2	14.2	50.5	22.1
Level of education					
Postgraduate or higher	769 (39)	6.9	16.6	54.9	21.6
Undergraduate	970 (50)	7.6	18.5	51.2	22.7
All others	221 (11)	12.2	12.7	46.2	29.0
Type of urban area of bicycle use					
Central municipality	450 (23)	6.7	11.3	55.8	26.2
Provincial capital	1092 (56)	7.3	18.7	52.6	21.4
Deputy provincial city	90 (5)	11.1	27.8	50.0	11.1
All others	328 (17)	10.4	16.8	46.0	26.8
Province/City of bicycle use					
Hunan	579 (30)	7.4	18.5	55.1	19.0
Guangdong	230 (12)	8.7	25.2	52.2	13.9
Beijing	164 (8)	7.9	10.4	62.2	19.5
Tianjin	144 (7)	7.6	9.0	50.0	33.3
All others	843 (43)	7.9	16.6	48.4	27.0
Reason for travel					
Commuting to work/school	1070 (55)	6.9	17.0	53.9	22.1
Entertainment	544 (28)	8.3	17.8	52.2	21.7
Physical exercise	180 (9)	11.7	16.7	40.0	31.7
Others	166 (9)	8.4	15.7	53.0	22.9
Riding hours per week					
<1 hour	240 (12)	4.2	20.4	51.7	23.8
1–2 hours	732 (37)	8.1	17.1	53.3	21.6
3–5 hours	877 (45)	7.6	17.1	51.3	23.9
>5 hours	111 (6)	16.2	9.9	51.4	22.5
Type of typical riding days					
Weekday	1105 (56)	7.2	16.1	54.4	22.3
Weekend or holiday	855 (44)	8.7	18.4	49.1	23.9
Typical riding time					
Morning rush hours	399 (20)	8.3	15.8	53.4	22.6
Evening rush hours	698 (36)	8.3	17.0	53.3	21.3
Other times	863 (44)	7.3	17.7	50.5	24.4

**Table 6:** Proportion of riders carrying passengers.

Variable	Number (%)	Frequency of behavior (%)			
		Always	Often	Sometimes	Never
Total	1960 (100)	0.5	0.7	4.3	94.6
Sex					
Male	833 (43)	0.6	0.7	5.6	93.0
Female	1127 (58)	0.4	0.6	3.3	95.7
Age group					
≤25 years	1056 (54)	0.2	0.8	3.3	95.7
26∼35 years	623 (32)	0.8	0.8	5.5	92.9
≥36 years	281 (14)	0.7	0.0	5.3	94.0
Level of education					
Postgraduate or higher	769 (39)	0.1	0.4	3.3	96.2
Undergraduate	970 (50)	0.3	0.7	3.7	95.3
All others	221 (11)	2.3	1.4	10.4	86.0
Type of urban area of bicycle use					
Central municipality	450 (23)	0.9	1.3	3.6	94.2
Provincial capital	1092 (56)	0.2	0.4	3.0	96.4
Deputy provincial city	90 (5)	0.0	0.0	2.2	97.8
All others	328 (17)	0.9	0.9	10.1	88.1
Province/City of bicycle use					
Hunan	579 (30)	0.5	0.7	3.6	95.2
Guangdong	230 (12)	0.0	0.0	5.2	94.8
Beijing	164 (8)	0.6	0.6	4.3	94.5
Tianjin	144 (7)	0.0	0.7	3.5	95.8
All others	843 (43)	0.6	0.8	4.6	94.0
Reason for travel					
Commuting to work/school	1070 (55)	0.5	0.6	3.6	95.3
Entertainment	544 (28)	0.6	0.9	5.1	93.4
Physical exercise	180 (9)	0.6	1.1	8.3	90.0
Others	166 (9)	0.0	0.0	1.2	98.8
Riding hours per week					
<1 hour	240 (12)	0.0	0.0	2.5	97.5
1–2 hours	732 (37)	0.3	0.7	4.1	94.9
3–5 hours	877 (45)	0.5	0.8	4.6	94.2
>5 hours	111 (6)	2.7	0.9	7.2	89.2
Type of typical riding days					
Weekday	1105 (56)	0.3	0.4	3.1	96.3
Weekend or holiday	855 (44)	0.7	1.1	5.8	92.4
Typical riding time					
Morning rush hours	399 (20)	0.5	0.3	6.8	92.5
Evening rush hours	698 (36)	0.4	0.7	4.3	94.6
Other times	863 (44)	0.5	0.8	3.1	95.6

**Table 7:** Proportion of riders using a cell phone while riding.

Variable	Number (%)	Frequency of behavior (%)			
		Always	Often	Sometimes	Never
Total	1960 (100)	1.2	4.2	37.6	57.0
Sex					
Male	833 (43)	1.8	6.5	44.5	47.2
Female	1127 (58)	0.8	2.5	32.4	64.3
Age group					
≤25 years	1056 (54)	0.9	5.1	38.7	55.2
26∼35 years	623 (32)	1.8	4.0	39.6	54.6
≥36 years	281 (14)	1.1	1.1	28.5	69.4
Level of education					
Postgraduate or higher	769 (39)	1.3	3.8	38.2	56.7
Undergraduate	970 (50)	0.8	4.8	36.9	57.4
All others	221 (11)	2.7	2.7	38.0	56.6
Type of urban area of bicycle use					
Central municipality	450 (23)	1.6	4.9	40.2	53.3
Provincial capital	1092 (56)	0.6	4.2	34.5	60.6
Deputy provincial city	90 (5)	2.2	4.4	35.6	57.8
All others	328 (17)	2.4	3.0	44.5	50.0
Province/City of bicycle use					
Hunan	579 (30)	0.9	2.6	32.0	64.6
Guangdong	230 (12)	1.3	4.3	33.0	61.3
Beijing	164 (8)	1.2	5.5	36.0	57.3
Tianjin	144 (7)	2.8	6.9	38.9	51.4
All others	843 (43)	1.2	4.5	42.7	51.6
Reason for travel					
Commuting to work/school	1070 (55)	1.2	5.4	39.3	54.0
Entertainment	544 (28)	1.5	3.5	35.8	59.2
Physical exercise	180 (9)	1.7	1.7	36.1	60.6
Others	166 (9)	0.0	1.2	33.1	65.7
Riding hours per week					
<1 hour	240 (12)	0.4	3.8	26.7	69.2
1–2 hours	732 (37)	1.1	3.8	41.8	53.3
3–5 hours	877 (45)	1.3	4.7	36.8	57.2
>5 hours	111 (6)	3.6	3.6	38.7	54.1
Type of typical riding days					
Weekday	1105 (56)	1.0	5.2	38.0	55.7
Weekend or holiday	855 (44)	1.5	2.8	37.0	58.7
Typical riding time					
Morning rush hours	399 (20)	1.3	4.0	41.4	53.4
Evening rush hours	698 (36)	1.9	3.9	36.5	57.7
Other times	863 (44)	0.7	4.5	36.6	58.2

**Table 8:** Proportion of riders eating while riding.

Variable	Number (%)	Frequency of behavior (%)			
		Always	Often	Sometimes	Never
Total	1960 (100)	0.8	1.4	20.1	77.8
Sex					
Male	833 (43)	1.1	2.8	25.0	71.2
Female	1127 (58)	0.5	0.4	16.5	82.6
Age group					
≤25 years	1056 (54)	0.6	1.6	20.9	76.9
26∼35 years	623 (32)	0.6	1.4	22.3	75.6
≥36 years	281 (14)	1.8	0.4	12.1	85.8
Level of education					
Postgraduate or higher	769 (39)	0.8	0.8	20.2	78.3
Undergraduate	970 (50)	0.4	1.9	20.7	77.0
All others	221 (11)	2.3	1.4	17.2	79.2
Type of urban area of bicycle use					
Central municipality	450 (23)	0.9	1.1	18.7	79.3
Provincial capital	1092 (56)	0.5	1.1	18.9	79.5
Deputy provincial city	90 (5)	0.0	3.3	18.9	77.8
All others	328 (17)	1.5	2.1	26.5	69.8
Province/City of bicycle use					
Hunan	579 (30)	0.5	1.2	18.5	79.8
Guangdong	230 (12)	1.3	1.3	17.4	80.0
Beijing	164 (8)	1.2	1.2	13.4	84.1
Tianjin	144 (7)	0.0	2.1	19.4	78.5
All others	843 (43)	0.8	1.4	23.4	74.4
Reason for travel					
Commuting to work/school	1070 (55)	0.9	1.1	21.0	76.9
Entertainment	544 (28)	0.6	2.0	20.2	77.2
Physical exercise	180 (9)	1.1	1.7	16.7	80.6
Others	166 (9)	0.0	0.6	17.5	81.9
Riding hours per week					
<1 hour	240 (12)	0.0	0.8	12.9	86.2
1–2 hours	732 (37)	0.5	1.4	23.1	75.0
3–5 hours	877 (45)	0.7	1.6	20.0	77.8
>5 hours	111 (6)	4.5	0.9	17.1	77.5
Type of typical riding days					
Weekday	1105 (56)	0.8	1.4	19.6	78.2
Weekend or holiday	855 (44)	0.7	1.4	20.7	77.2
Typical riding time					
Morning rush hours	399 (20)	1.0	0.5	20.8	77.7
Evening rush hours	698 (36)	1.0	2.1	19.8	77.1
Other times	863 (44)	0.5	1.2	20.0	78.3

## 2 Experimental design, materials and methods

### 2.1 Study recruitment and participants

Almost all shared bicycles in urban China are rented from smartphone applications [2], so we used an iterative sampling process to recruit study participants through WeChat, the most popular smartphone-based social media program in China. Non-probability sampling has advantages in recruiting study samples compared to probability sampling when random samples are unlikely to be obtained [3]. Initial survey invitations were sent to a convenience sample of colleagues, family members, classmates, and friends who had WeChat contact with members of the research group. Many of these individuals chose to participate, and they then were asked to send information about the survey to people in their own WeChat contact list. This “snowball” recruitment process was iterated for a month, from September 7, 2017 to October 6, 2017, at which point the sample size was deemed sufficient and data collection was terminated.

In total, 1960 riders participated in the retrospective research survey. Of them, individuals aged ≤25 years old, 26–35 years old, and ≥36 years accounted for 54%, 32%, and 14% of participants, respectively. Males constituted 43% of participants. 50% and 39% of respondents respectively reported having received an undergraduate degree and postgraduate education or higher. The majority of respondents came from provincial capitals (56%) and central municipalities (23%). Geographically, the participants came primarily from Hunan Province (29.5%), Guangdong Province (11.7%), Beijing city (8.4%) and Tianjin city (7.3%), with the remainder spread across China.

### 2.2 Questionnaire

The questionnaire, which included three parts, was designed based on previous epidemiological surveys and empirical information from media reports. The first part of the survey questionnaire included variables concerning demographic traits (sex, age, level of education, type of city where they lived and rode shared bicycles). The second part consisted of shared bicycle travel-related information, such as typical purpose of shared bicycle travel, number of shared bicycle riding hours a week, and riding time for average shared bicycle rides. The third and final part of the survey asked about frequency of engaging in eight unsafe shared bicycle riding behaviors: (1) not wearing helmets [4], [5], [6], [7], (2) running red lights [8], (3) cycling against the traffic flow [9], (4) riding in a motor vehicle lane where bicycles are prohibited, (5) riding in a pedestrian lane where bicycles are prohibited, (6) carrying passengers on a shared bicycle with only one seat [10], (7) using a cell phone while riding a shared bicycle, and (8) eating while riding a shared bicycle [11], [12]. The eight risky behaviors were developed through a series of steps involving a thorough review of existing research literature and media reports, multi-round group discussions among the research team, and pilot testing. Participants responded to each survey item by identifying the frequency with which they engaged in each behavior over the past month using a 4-point scale (always, often, sometimes, never).

### 2.3 Statistical analysis

Data analysis involved computation of basic descriptive statistics presenting the frequency of each of the eight unsafe riding behaviors, which were derived through participant self-report. SPSS (Statistical Product and Service Solutions) statistical software version 22.0 (IBM Corp, Armonk, NY, US) was used to perform all statistical analyses.
