# Supplementary material for: Data on unsafe riding behaviors among 1960 shared bicycle riders in urban China
Source: Data Brief. 2019 Jul 27;25:104329. doi: 10.1016/j.dib.2019.104329 (PMC6700483; doi:10.1016/j.dib.2019.104329)
Supplement: Supplementary file 1 [file mmc1.docx]

**Questionnaire on the circumstances of the shared bicycle users**

***Section 1 Personal information***

**Question 1: What city and region are you located in?**

The answer options included the detail provinces, cities, and counties or districts.

Answer: (the drop-down option)

**Question 2: What is the type of your city and region where you are located?**

A. Central municipality

B. Provincial capital

C. Deputy provincial city

D. Prefecture city

E. County

F. Town

**Question 3: What’s your gender?**

A. Male

B. Female

**Question 4: What’s your age?**

Answer:

**Question 5: What’s your level of education?**

A. Junior high or lower

B. Senior high

C. Junior college graduate

D. Undergraduate

E. Postgraduate or higher

***Section 2 Shared bicycle riding behaviours-related***

**Question 6: When do you typically ride shared bicycles?**

A. 7:30-8:30

B. 9:30-10:30

C. 11:30-12:30

D. 15:00-16:00

E. 17:00-18:00

F. Other times

**Question 7: During what type of day do you typically ride shared bicycles?**

A. Weekdays

B. Weekends

C. Holidays

**Question 8: What is your main purpose for riding shared bicycles?**

A. Commuting to work/school

B. Entertainment

C. Physical exercise

D. Other (Please provide details to explain.)

**Question 9: How many hours per week do you ride shared bicycles, on average?**

Answer:

**Question 10: Did you ever engage in the following behaviours while riding shared bicycles in the past month?**

A. Wearing helmets 🞏 Always 🞏 Often 🞏 Sometimes 🞏 Never

B. Running red lights 🞏 Always 🞏 Often 🞏 Sometimes 🞏 Never

C. Cycling against the traffic flow 🞏 Always 🞏 Often 🞏 Sometimes 🞏 Never

D. Riding in a motor vehicle lane 🞏 Always 🞏 Often 🞏 Sometimes 🞏 Never

E. Riding in a pedestrian lane 🞏 Always 🞏 Often 🞏 Sometimes 🞏 Never

F. Carrying passengers 🞏 Always 🞏 Often 🞏 Sometimes 🞏 Never

G. Using a cell phone while riding 🞏 Always 🞏 Often 🞏 Sometimes 🞏 Never

H. Eating while riding 🞏 Always 🞏 Often 🞏 Sometimes 🞏 Never

**Note:**

The raw data related to the frequency of each of the eight unsafe riding behaviours that participants self-reported are shown in *Supplementary file_2*.
